# Airway Microbiota in Bronchoalveolar Lavage Fluid from Clinically Well Infants with Cystic Fibrosis

**DOI:** 10.1371/journal.pone.0167649

**Published:** 2016-12-08

**Authors:** Theresa A. Laguna, Brandie D. Wagner, Cynthia B. Williams, Mark J. Stevens, Charles E. Robertson, Cole W. Welchlin, Catherine E. Moen, Edith T. Zemanick, Jonathan K. Harris

**Affiliations:** 1 Department of Pediatrics, University of Minnesota School of Medicine and the Masonic Children’s Hospital, Minneapolis, Minnesota, United States of America; 2 Department of Biostatistics and Informatics, Colorado School of Public Health, University of Colorado Denver, Aurora, Colorado, United States of America; 3 Department of Pediatrics, University of Colorado School of Medicine, Aurora, Colorado, United States of America; 4 Department of Medicine, University of Colorado School of Medicine, Aurora, Colorado, United States of America; University of Illinois at Urbana-Champaign, UNITED STATES

## Abstract

**Background:**

Upper airway cultures guide the identification and treatment of lung pathogens in infants with cystic fibrosis (CF); however, this may not fully reflect the spectrum of bacteria present in the lower airway. Our objectives were to characterize the airway microbiota using bronchoalveolar lavage fluid (BALF) from asymptomatic CF infants during the first year of life and to investigate the relationship between BALF microbiota, standard culture and clinical characteristics.

**Methods:**

BALF, nasopharyngeal (NP) culture and infant pulmonary function testing data were collected at 6 months and one year of age during periods of clinical stability from infants diagnosed with CF by newborn screening. BALF was analyzed for total bacterial load by qPCR and for bacterial community composition by 16S ribosomal RNA sequencing. Clinical characteristics and standard BALF and NP culture results were recorded over five years of longitudinal follow-up.

**Results:**

12 BALF samples were collected from 8 infants with CF. *Streptococcus*, *Burkholderia*, *Prevotella*, *Haemophilus*, *Porphyromonas*, and *Veillonella* had the highest median relative abundance in infant CF BALF. Two of the 3 infants with repeat BALF had changes in their microbial communities over six months (Morisita-Horn diversity index 0.36, 0.38). Although there was excellent percent agreement between standard NP and BALF cultures, these techniques did not routinely detect all bacteria identified by sequencing.

**Conclusions:**

BALF in asymptomatic CF infants contains complex microbiota, often missed by traditional culture of airway secretions. Anaerobic bacteria are commonly found in the lower airways of CF infants.

## Introduction

Progressive, obstructive lung disease remains the biggest cause of morbidity and early mortality in cystic fibrosis (CF). Mucus stasis and impaired bacterial killing provide the optimal environment for chronic bacterial infection in the CF airway, with previous work identifying a small number of “traditional pathogens” thought to drive the structural damage and loss of lung function characteristic of CF [1–4]. Standard-of-care requires a quarterly airway culture from all CF patients, seeking to identify treatable bacteria that may alter the airway environment and lead to more aggressive lung disease. However, this targeted view of bacterial infection and CF lung disease does not reveal the full extent of polymicrobial communities in the airway, given traditional culture is limited in its scope. Even more limiting is the fact the infants and young children often do not expectorate sputum, making an upper airway culture the accepted surrogate for what bacteria may be found in the lower airways. Culture-independent approaches using next-generation sequencing techniques in infants and children have revealed a more complex, polymicrobial community in the CF upper airway than previously identified using traditional culture, although determining which microbiota residing in the upper airway are present in the lower airway has proved to be challenging [5,6].

Although we continue to gain knowledge about the relative abundance of bacteria and the temporal stability of the microbiota of the CF airway, significant gaps in knowledge remain, specifically in the pediatric population. Younger patients with CF tend to have higher diversity in their airway microbiota that progressively decreases with age and as obstructive lung disease worsens, although most studies to date have relied on upper airway samples [oropharyngeal (OP) or nasopharyngeal (NP)] and cross sectional data [7–11]. Although bronchoscopy with bronchoalveolar lavage provides a lower airway sample with limited opportunities for upper airway contamination, surveillance bronchoscopy in infants with CF is not routinely performed and infants and young children with CF often do not expectorate sputum, making studies of lower airway microbiota in this population challenging [12–14]. Recent work utilizing 16S ribosomal RNA (rRNA) gene sequencing in OP and NP samples from CF infants and healthy controls has characterized the microbiota present in the upper airway [5,6]. Although this work provides important insight into bacteria present in the CF infant upper airway, neither utilized a lower airway sample [i.e. bronchoalveolar lavage fluid (BALF)] to determine if these bacteria are truly present in the lower airways. Information on the lower airway microbiota in this largely asymptomatic population of CF infants and the clinical significance of these findings remains unknown.

We performed a prospective cohort study using 16S rRNA sequencing to characterize the microbiota present in BALF from a small cohort of asymptomatic infants with CF during the first year of life. We collected a BALF culture, an NP culture and infant pulmonary function (iPFT) testing data at 6 months and one year of age. We utilized up to five years of standard upper airway surveillance culture and clinical outcome data from these subjects from our internal CF Center database. Our objectives were to describe the microbiota of BALF from asymptomatic CF infants during the first year of life and to describe the relationship between BALF microbiota, standard BALF and NP culture and clinical characteristics at up to five years of longitudinal follow-up. Some of the results of these studies have been previously reported in the form of an abstract [15].

## Materials and Methods

### Study Design and Subjects

A single-center, longitudinal, one-year prospective cohort study was performed. Infants diagnosed with CF via the Minnesota State Newborn Screening Program were recruited and enrolled at their 3 month outpatient clinic visit to the Minnesota CF Center from 2009–2010. Infants were required to have a confirmed diagnosis of CF as evidence by sweat chloride ≥ 60 mmol Cl^−^/L and two CFTR disease-causing mutations. We collected demographic data, measured blood and airway inflammatory markers, performed bronchoscopy with lavage, obtained BALF and NP cultures and performed infant pulmonary function testing (iPFT) at six months and one year of age. Our Minnesota CF Center internal database provided NP culture and clinical outcome data for up to five years of longitudinal follow-up after the initial bronchoscopy. The University of Minnesota Masonic Children’s Hospital moved to a new facility and leadership of the Pediatric Sedation Unit changed during study enrollment. This move necessitated bronchoscopies with lavage in infants be performed in the operating room under general anesthesia, a procedure that was beyond the budget of the original grant funding this protocol, thus limiting enrollment. The University of Minnesota (UMN) Institutional Review Board approved the study protocol and written informed consent was obtained from each of the subject’s parents or legal guardians.

### Procedures and Specimen Collection

#### Infant Lung Function Analysis

Infant pulmonary function testing is performed in the Pediatric Sedation Unit by the Minnesota CF Center every 6 months for the first two years of life as standard of care. For the iPFT procedure, infants were sedated with oral chloral hydrate (100 mg/kg) and oral hydroxyzine (0.5 mg/kg). Pulmonary function assessments included measurements of forced expiratory volumes [forced expiratory volume in 0.5 seconds (FEV0.5) and forced vital capacity (FVC)] by the raised volume rapid thoracoabdominal compression technique [16–18].

#### BALF and NP Specimen Collection

To use the sedation previously provided, the bronchoscopy with lavage was performed immediately upon completion of the iPFT procedure in the Pediatric Sedation Unit. No additional sedation was administered to infants, so bronchoscopy was not performed if additional sedation was required. A single physician performed flexible fiberoptic bronchoscopy with bronchoalveolar lavage in the right middle lobe of CF infants. All bronchoscopies were performed intra-nasally and suction channel use was avoided until the bronchoscope tip extended beyond the carina. Two aliquots of 1mL/kg of sterile normal saline were instilled into the right middle lobe. All BALF was pooled and immediately placed on ice. Nasopharyngeal specimens were collected before each bronchoscopy procedure by inserting a suction catheter through the nose followed by gentle suction. The specimen was immediately sent to the clinical laboratory at the UMN for microbiological analysis.

#### Sample Processing

The NP suction specimen and one 5 mL aliquot of BALF was sent to the UMN clinical laboratory for cell count and differential and the qualitative identification of respiratory pathogens by standard microbiological techniques [4,19,20]. The remaining BALF sample was centrifuged at 250 x g for 10 minutes at 4°C. The supernatant was transferred with a sterile pipette to a separate tube and 0.5 mL sterile saline was added to the remaining pellet and stored at -80°C. No protease inhibitors were added. This pellet was shipped on dry ice to the Pediatric Microbiome Laboratory at the University of Colorado for microbiota analysis.

### Laboratory Assays

#### High-throughput DNA sequencing for microbiota analysis

DNA extractions were performed using the Qiagen EZ1 Advanced automated extraction platform (Qiagen Inc., Valencia, CA) with the Tissue Kit and Bacterial card per manufacturer's instructions. Total bacterial load (TBL) (gene copies/reaction) was estimated for each sample and negative control blanks using a TaqMAN assay [21,22]. Two PCR negative controls were included on the same TBL plate used for these samples, the blanks returned values of 0 and 36.6 copies/reaction. The higher value was used for comparison to the TBL values from the samples and corresponds to 6.3 log_10_ copies/mL after multiplying by a 50,000 dilution factor. *16S Amplicon Library Construction*: Bacterial profiles were determined by broad-range amplification and sequence analysis of 16S ribosomal RNA (rRNA) genes following our previously described methods [23,24]. Amplicons were generated using MiSeq compatible primers that target approximately 300 base pairs of the V1V2 variable region (27F/338R) of the 16S rRNA gene. A negative control reaction was run for each sample (unique paired barcode combination) to ensure unintended DNA input was not responsible for any amplification products observed. None of the negative control reactions resulted in a visible band on an agarose gel. Polymerase chain reaction (PCR) products were normalized using agarose gel densitometry, pooled, purified and concentrated using a DNA Clean and Concentrator Kit (Zymo, Irvine, CA). Pooled amplicons were quantified using Qubit Fluorometer 2.0 (Invitrogen, Carlsbad, CA). The pool was diluted to 4nM and denatured with 0.2 N NaOH at room temperature. The denatured DNA was diluted to 20pM and spiked with 10% of the Illumina PhiX control DNA prior to loading the sequencer. Illumina paired-end sequencing was performed on the Miseq platform using a 500 cycle version 2 reagent kit. *Analysis of Illumina Paired-end Reads*: Illumina Miseq paired-end reads were aligned to human reference genome Hg19 with bowtie2 and matching sequences discarded [25]. As previously described, the remaining non-human paired-end sequences were sorted by sample via barcodes in the paired reads with a python script [24]. Sorted paired end sequence data were deposited in the NCBI Short Read Archive under accession number SRP066906. The sorted paired reads were assembled using phrap [26,27]. Pairs that did not assemble were discarded. Assembled sequence ends were trimmed over a moving window of 5 nucleotides until average quality met or exceeded 20. Trimmed sequences with more than 1 ambiguity or shorter than 200 nt were discarded. Potential chimeras identified with Uchime (usearch6.0.203_i86linux32) [28] using the Schloss [29] Silva reference sequences were removed from subsequent analyses. Assembled sequences were aligned and classified with SINA (1.2.11) [30] using the 418,497 bacterial sequences in Silva 115NR99 [31] as reference configured to yield the Silva taxonomy. Operational taxonomic units (OTUs) were produced by clustering sequences with identical taxonomic assignments. This process generated 390,248 sequences for 11 samples (average sequence length: 316 nt; average sample size: 35,477 with a range 18,043 to 60,288 sequences). 225 taxa were identified across the 11 samples. The median Goods coverage score was ≥ 99.85% at the rarefaction point of 18,043. The software package Explicet (v2.10.5, www.explicet.org) was used for display and calculation of common sequencing measures (rarefied values for median Good’s coverage and Shannon alpha diversity) [32].

#### BALF Inflammatory Markers

BALF neutrophil elastase was quantified by a spectrophotometric assay based on the hydrolysis of the specific substrate MeO-suc-Ala-Ala-Pro-Ala-p-nitroanilide (Sigma Chemical Co.; St. Louis, MO) and interleukin-8 concentration was measured as part of an eight-plex panel (EMD Millipore Corporation; Millerica, MA).

### Statistical Analysis

Descriptive statistics include the median and range for all variables. To account for differences in sequencing depth, the relative abundance (RA) of each genus was calculated (number of sequences for specific genera/total number of sequences *100). Shannon alpha diversity index, richness and evenness, and Morisita-Horn (MH) beta diversity [33] were calculated in Explicet [32]. Percent agreement between BALF and NP culture was calculated to compare detection rates of both sample types under the assumption that neither approach is the gold standard for comparison. Pearson correlation coefficients were used to determine the correlation between microbial markers (for taxa with at least a 5% relative abundance in a sample) and clinical variables (*i*.*e*. lung function, age). All analyses were performed using SAS version 9.4 software (SAS Institute Inc.: Cary, NC, 2013).

## Results

### Subject Characteristics

Demographic data from 8 infants with CF, involving 12 BALF samples, are presented in Table 1. All infants were asymptomatic and were not taking antibiotics at the time of the initial bronchoscopy. We were unable to obtain the second BALF sample on four infants secondary to inadequate sedation after the iPFT to perform the bronchoscopy. Our subjects had a median (range) FEV_0.5_ of 94% predicted (69% - 116%). The distribution of genotypes in the study cohort was predominantly F508del homozygous and all were pancreatic insufficient. See S1 Table for additional clinical data for each subject at each time point in the study.

**Table 1:** Subject demographics.

Characteristics	First BALF	Second BALF
Subjects	8	3
BALF samples	8	3
Female:Male	6:2	3:0
Age at BAL (months)	6.8 (5.7–11.8)	12.4 (11.9–13.2)
FEV_0.5_ at BAL (% predicted)	94 (69–116)	124 (97–145)
F508del/F508del	7 (88%)	3 (100%)
F508del/G551D	1 (12%)	0

Values are presented as number (%) or median (range).

### Description of Microbial Communities

#### Cross-Sectional BALF Samples

**Fig 1:**
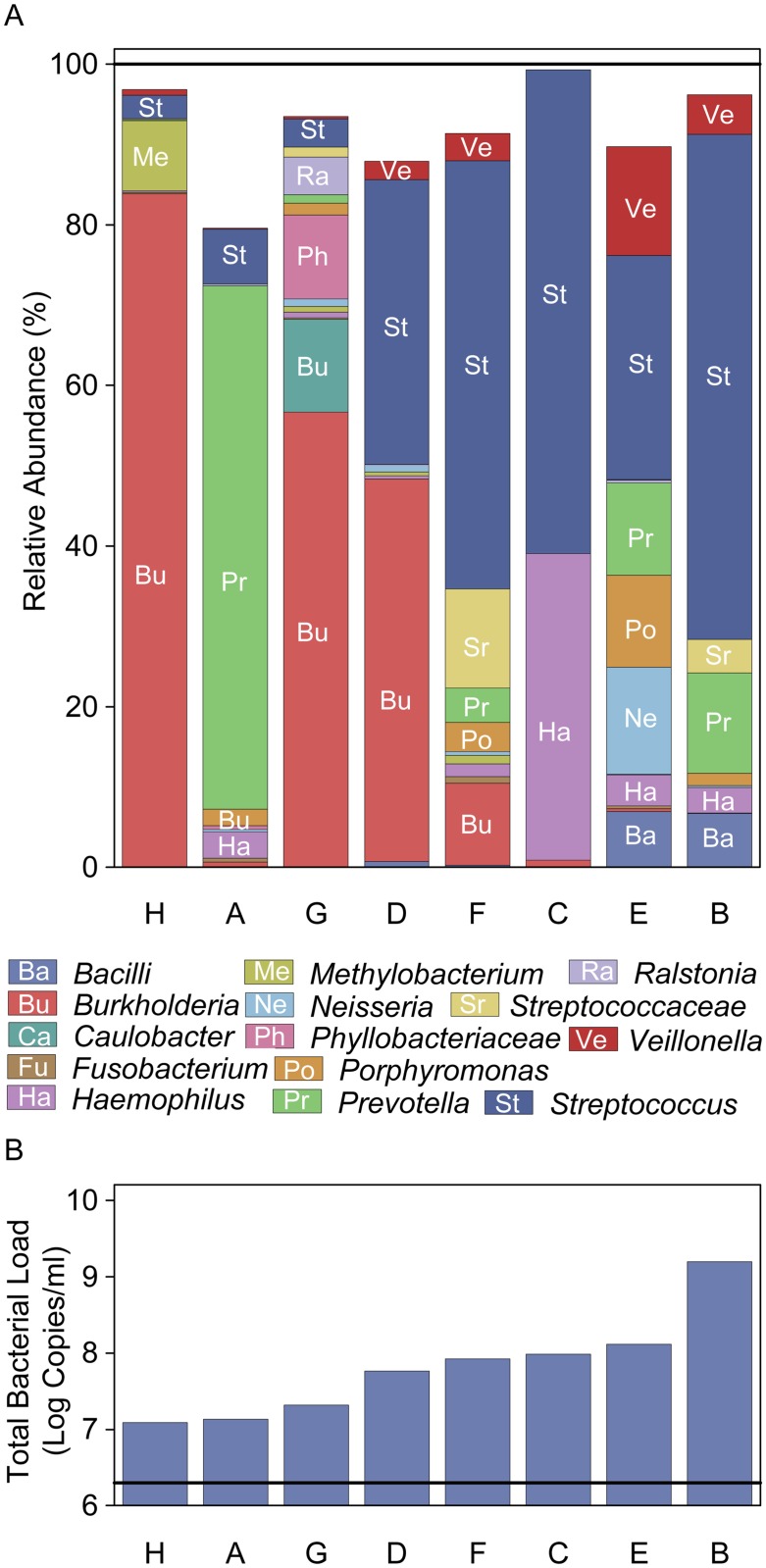
Total bacterial load and sequencing results from CF infant BALF samples. (A) The top graph shows the relative abundance (RA) of specific taxon identified in the eight cross-sectional BALF samples. In specimens where total RA displayed is < 100%, the proportion not displayed was made up of other taxa present in low RA (< 5%). The samples are ordered by total bacterial load (TBL) detected in each sample. (B) The lower graph shows the TBL detected from each sample. The black horizontal line indicates the value obtained from one of the two negative controls included on the TBL qPCR plate, the other control produced a 0 value. Each letter represents an individual patient. Each taxon was required to have a RA of > 5% in at least 1 sample to be represented.

The median (range) total bacterial load (TBL) for the eight cross-sectional BALF samples was 7.8 log_10_ rDNA copies/mL (7.1–9.2). Eleven of the twelve (92%) BALF pellets had successful amplification for sequencing, with one pellet having too low of a TBL to permit amplification. The median (range) Shannon Diversity Index was 2.2 (1.1–3.2). The median (range) for richness was 70.9 (17.1–92.6). The median (range) for the evenness in the BALF samples was 0.4 (0.2–0.6). Two hundred twenty-three taxa were detected across all the samples with the 10 taxa present in the greatest relative abundance in the 8 cross-sectional BALF samples displayed in Table 2. The taxa *Streptococcus*, *Burkholderia*, *Prevotella*, *Haemophilus*, *Veillonella*, *Gemella*, *Neisseria* and *Methylobacterium* were detected in all 8 initial BALF samples. The RA of taxa and the corresponding TBL for each of the 8 initial BALF samples from infants with CF is displayed in Fig 1A and 1B). *Burkholderia* is the predominant taxa in three samples and *Prevotella* was dominant in one sample.

**Table 2:** The top 10 taxa with the highest mean relative abundance across the 8 cross-sectional BALF samples.

Phyla	Taxa	n	% samplestaxadetected	MedianRA	lIQR	uIQR	Min	Max
**Firmicutes**	**Streptococcus**	**8**	**100.0**	**31.69**	**5.15**	**56.75**	**2.94**	**62.86**
Proteobacteria	Burkholderia	8	100.0	5.56	0.50	52.16	0.04	83.86
**Bacteroidetes**	**Prevotella**	**8**	**100.0**	**2.65**	**0.04**	**12.02**	**0.01**	**65.16**
Proteobacteria	Haemophilus	8	100.0	2.38	0.56	3.59	0.17	38.17
**Bacteroidetes**	**Porphyromonas**	**8**	**75.0**	**1.50**	**0.01**	**2.85**	**0.00**	**11.46**
**Firmicutes**	**Veillonella**	**8**	**100.0**	**1.49**	**0.20**	**4.19**	**0.01**	**13.59**
Firmicutes	Gemella	8	100.0	0.70	0.09	2.35	0.00	4.36
Proteobacteria	Neisseria	8	100.0	0.39	0.13	0.93	0.00	13.36
Firmicutes	Lactobacillales	8	87.5	0.32	0.13	1.41	0.00	2.78
Proteobacteria	Methylobacterium	8	100.0	0.22	0.01	0.90	0.00	8.73

RA = Relative abundance; lIQR = lower interquartile range; uIQR = upper interquartile range. COLOR = anaerobic community.

#### Longitudinal BALF Samples

**Fig 2:**
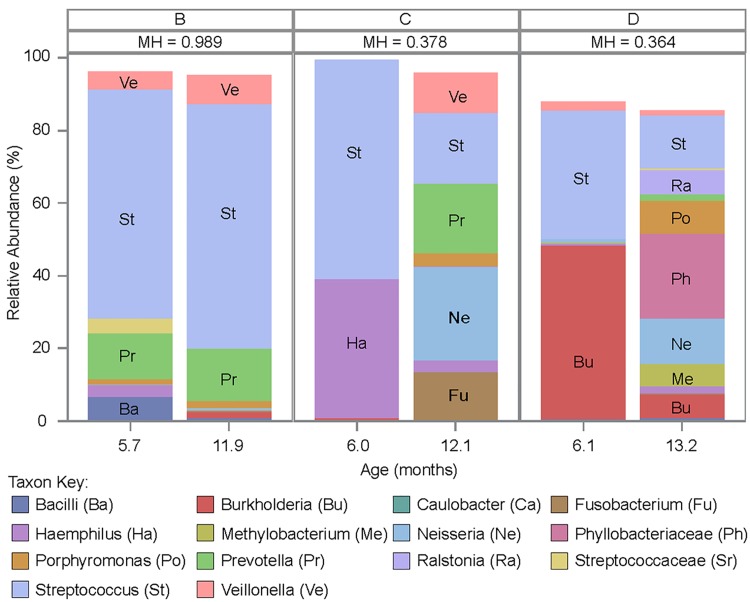
Comparison of the BALF microbiota communities in CF infants with repeat BALF performed 6 months apart. Two BALF pairs had large shifts in their microbial communities as represented by a MH index of < 0.38. Each letter represents an individual patient.

Two BALF samples were collected from 4 subjects approximately 6 months apart (Fig 2). One of these samples did not contain enough 16S rRNA gene copies for sequencing so the comparison was made on the three remaining pairs. The MH beta diversity index is a community level similarity measure which ranges from 0 to 1, with 1 indicating identical bacterial communities [33]. Two of the three sample pairs had a MH index ≤ 0.38, indicating shifts in the microbial communities between the first and second BALF. One of these two sample pairs was on tobramycin inhalation solution at the time of the second bronchoscopy; the other had not received any oral or inhaled antibiotics between procedures. The final sample pair had a nearly identical bacterial community (MH ~ 1).

### Comparison of Microbiota with Standard BALF and NP Culture

Table 3 displays the comparison of the BALF culture, NP culture and 16S rRNA sequencing for detecting traditional CF-related bacterial pathogens in the upper and lower airway of infants with CF (*i*.*e*. *Pseudomonas*, *Staphylococcus*, *Haemophilus*, *Stenotrophomonas* and *Achromobacter*). All eleven BALF samples were used for this analysis. 16S rRNA sequencing detected the genera *Pseudomonas* in 55% of the samples (6/11), *Staphylococcus* in 100% (11/11), *Haemophilus* in 100% (11/11), *Stenotrophomonas* in 73% (8/11) and *Achromobacter* in 27% (3/11). Of those samples with CF-related bacterial organisms detected by sequencing, the BALF culture detected *P*. *aeruginosa* in only 15% (1/6), *S*. *aureus* in 36% (4/11), *H*. *influenzae* in 9% (1/11), and did not detect *S*. *maltophilia* or *A*. *xylosoxidans*. The percent agreement between BALF culture and NP culture was 90% for *P*. *aeruginosa*, *S*. *aureus* and *S*. *maltophilia* (10/11), 82% for *H*. *influenzae* and 100% for *A*. *xylosoxidans*. The NP culture was positive and identified the same organism detected by sequencing four times when the BALF culture failed to identify an organism present in the lower airway [*Pseudomonas* (1), *Staphylococcus* (1), and *Haemophilus* (2)]. One NP culture was positive for *S*. *maltophilia* when both sequencing and BALF culture failed to identify any bacteria.

**Table 3:** Comparison of BALF culture, BALF sequencing and NP culture for the identification of five common CF bacterial pathogens.

SID	Age at BAL (months)	Pseudomonas			Staphylococcus			Haemophilus			Stenotrophomonas			Achromobacter		
		BALF cult	BALF seq	NP cult	BALF cult	BALF seq	NP cult	BALF cult	BALF seq	NP cult	BALF cult	BALF seq	NP cult	BALF cult	BALF seq	NP cult
A	5.7	**-**	0.01	**-**	**+**	4.66	**+**	**-**	3.29	**-**	**-**	0.01	**-**	**-**	**-**	**-**
B	5.7	**+**	<0.01	**+**	**+**	0.18	**+**	**-**	3.20	**-**	**-**	**-**	**+**	**-**	**-**	**-**
B	11.9	**-**	**-**	**-**	**-**	0.01	**-**	**-**	0.13	**-**	**-**	<0.01	**-**	**-**	**-**	**-**
C	6.0	**-**	**-**	**-**	**+**	0.02	**+**	**+**	38.17	**+**	**-**	0.02	**-**	**-**	**-**	**-**
C	12.1	**-**	**-**	**-**	**-**	<0.01	**-**	**-**	3.36	**+**	**-**	**-**	**-**	**-**	**-**	**-**
D	6.1	**-**	**-**	**-**	**-**	0.53	**-**	**-**	0.44	**-**	**-**	0.42	**-**	**-**	**-**	**-**
D	13.2	**-**	0.07	**-**	**+**	0.94	**+**	**-**	1.76	**-**	**-**	0.36	**-**	**-**	**-**	**-**
E	6.2	**-**	**-**	**-**	**-**	0.08	**-**	**-**	3.89	**-**	**-**	**-**	**-**	**-**	**-**	**-**
F	6.5	**-**	0.01	**-**	**-**	0.05	**+**	**-**	1.57	**-**	**-**	0.04	**-**	**-**	<0.01	**-**
G	6.5	**-**	0.04	**+**	**-**	0.31	**-**	**-**	0.59	**-**	**-**	0.09	**-**	**-**	0.75	**-**
H	11.8	**-**	0.01	**-**	**-**	0.16	**-**	**-**	0.17	**+**	**-**	0.05	**-**	**-**	0.02	**-**

Each letter represents an individual patient. + = the bacteria was identified by standard culture;— = no growth was observed; BALF sequencing = % relative abundance of the specific genera in BALF. Culture is performed to the species level while sequencing is performed to the genus level.

### Relationships between CF BALF microbiota, clinical characteristics, and airway inflammation

**Fig 3:**
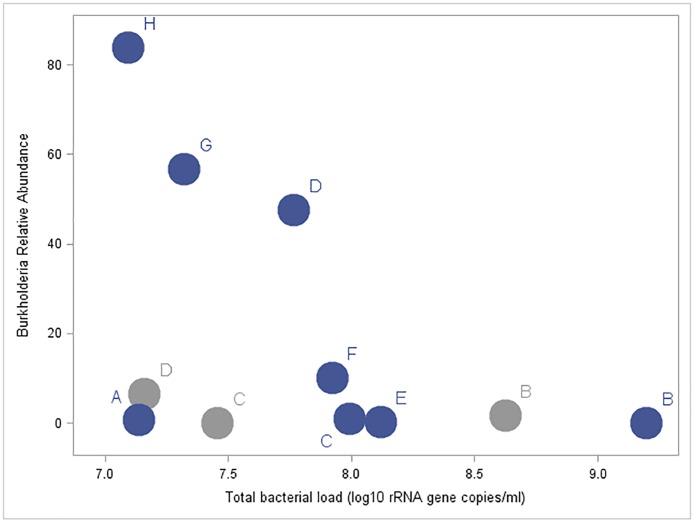
The three samples with larger RA of *Burkholderia* had low TBL values in CF infant BALF samples. Dark circles represent bronchoscopies performed at 6 months and light circles represent those performed at 12 months. Each letter represents an individual patient.

**Fig 4:**
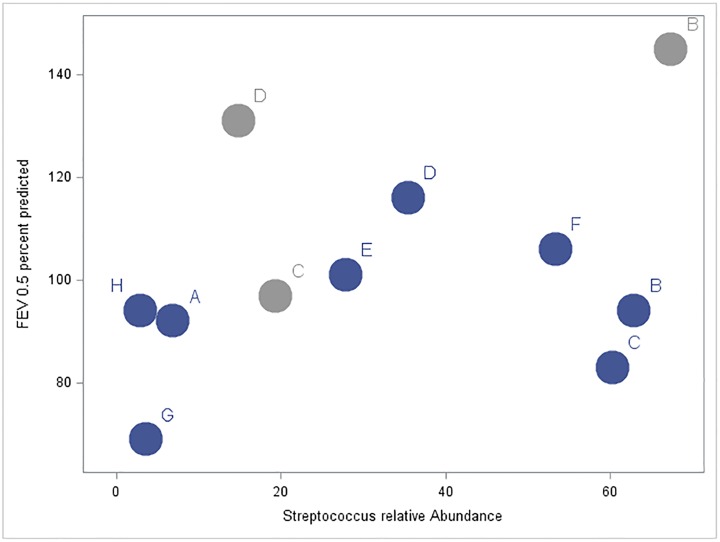
A positive correlation was observed between an increased RA of *Streptococcus* and a higher FEV_0.5_% predicted was observed in our CF infants during the first year of life. Dark circles represent bronchoscopies performed at 6 months and light circles represent those performed at 12 months. Each letter represents an individual patient.

There was no indication of an association between BALF neutrophil count with TBL (r = -0.13) or Shannon Diversity Index (r = -0.10). Given the presence of *Burkholderia* in multiple asymptomatic infant BALF samples, we performed a focused analysis on these samples to explore the possibility of *Burkholderia* being a pathogenic organism. Specifically, the three BALF samples with *Burkholderia* present on sequencing had lower TBL (Fig 3), did not have an increased BALF neutrophil count and these infants did not have a lower FEV_0.5_% predicted compared to other CF subjects. None of the infants had *Burkholderia* detected by standard culture. In addition, we observed a positive correlation between the RA of *Streptococcus* in BALF and FEV_0.5_% predicted during the first year of life with a correlation coefficient of 0.37 (95% CI = -0.47, 0.84) (Fig 4). All eight subjects had standard NP culture data available ranging from 21.6–61.3 months after the initial bronchoscopy. Two out of the 8 subjects went on to have a positive upper airway culture for *P*. *aeruginosa* and 7 out of the 8 subjects had at least one positive upper airway culture for *S*. *aureus*.

## Discussion

In this single center longitudinal study, we had the unique opportunity to study BALF obtained from asymptomatic infants with CF during the first year of life. We determined that BALF in this small cohort of infants contains complex and diverse microbiota identified by16S rRNA sequencing, often missed by traditional culture of upper airway secretions. We describe the most predominant taxa present in BALF in infants with CF and determined TBL and ecological characteristics, providing important information regarding lower airway microbiota in infants with CF. Surprisingly, we found anaerobic taxa to be present in all initial BALF samples, generating questions regarding the potential role of these organisms in the development of CF lung disease for future study.

Given previous studies of the microbiota of the CF infant airway in the first year of life have largely utilized upper airway samples, our use of infant BALF provides the first glimpse into the polymicrobial communities harbored in the lower airways. The most abundant taxa identified in our CF infant BALF samples were *Streptococcus*, *Burkholderia*, *Prevotella*, *Haemophilus*, *Veillonella*, *Gemella*, *Neisseria* and *Methylobacterium*. CF-associated pathogens such as *Pseudomonas*, *Stenotrophomonas* and *Staphylococcus* were also identified, but were less prevalent. While the presence of *Burkholderia* in our BALF samples is alarming, the samples that harbored the highest RA of *Burkholderia* had lower TBL and were closer to our negative control. This raises the possibility that the *Burkholderia* was not present in the airways, but was introduced during sample processing or handling. In addition, none of our CF infants were sick at the time of their bronchoscopy and none had a positive BALF or upper airway culture for *Burkholderia*, emphasizing the low bacterial burden of *Burkholderia* present in these samples. Our analysis did not reveal increased neutrophilic inflammation in the BALF of those infant samples with *Burkholderia* and the FEV_0.5_% predicted in these infants was not significantly lower than their counterparts. However, we did not utilize a normal saline lavage control, so we cannot say with complete certainty that *Burkholderia* was not present in the lower airway samples of these infants, but it is likely these sequences are not derived from the airways of these infants. *Burkholderia* has been found in previous microbiota work of the healthy and CF airway and this finding should be investigated in future studies [11,34].

Our current knowledge of the microbiota of the CF infant airway is largely from OP and NP swabs [5,6,35]. A core group of taxa—*Veillonella*, *Streptococcus*, *Bifidobacterium* and *Bacteroides* were identified in oropharyngeal and stool samples in a CF cohort from birth to 34 months, with microbial diversity increasing over time [5,35]. Prevaes et al. reported an NP microbial colonization profile in CF infants (that included bacteria regarded as non-pathogenic commensals) that differed from healthy controls and shifted over the first 6 months of life, likely influenced largely by antibiotic prophylaxis, a clinical practice not widely found in the United States [6]. Our study also documented a bacterial community shift in 2 out of three infants who had a repeat bronchoscopy, suggesting that the bacteria present in the lower airways is indeed dynamic and may not be secondary to antibiotic use alone. Granted our numbers were small; however, they generate interesting hypotheses regarding how the microbiota may evolve with time in CF. Our work now provides evidence that infants with CF less than a year of age may have a higher abundance of facultative anaerobic bacteria in their lower airways, the clinical significance of which should be determined with future study.

When compared to findings in the microbiota of lower airway samples (*i*.*e*. expectorated sputum and BALF) from older cohorts of children with CF, our work allows for some interesting comparisons. Coburn et al. identified a similar core microbiota of five genera–*Streptococcus*, *Prevotella*, *Rothia*, *Veillonella* and *Actinomyces* utilizing 16S ribosomal RNA sequencing in expectorated sputum from CF patients [11]. Utilizing 16s rRNA microarray technology in BALF, Renwick et al. found the *Proteobacteria*, *Firmicutes*, *Bacteroidetes* and *Tenericutes* phyla dominated the microbial community of the CF airway, similar to our findings in CF infants. While work in older CF patients suggests lower community diversity is associated with increased exposure to antibiotics and with lower lung function, our young CF patients had evidence of higher microbial diversity over time [11,36,37].

Sequencing detected significantly more bacterial taxa than were identified using traditional culture techniques of BALF and NP samples in our population, which is not a surprising finding. It is important to remember that next generation sequencing of 16S rRNA sequencing does not routinely identify bacterial DNA to the level of the species. Therefore, it is possible that *Pseudomonas* identified by sequencing is not *P*. *aeruginosa*. However, our findings continue to highlight the presence of *Streptococcus*, *Burkholderia*, *Prevotella*, *Haemophilus*, *Veillonella*, *Gemella*, *Neisseria* and *Methylobacterium* in the airway, none of which are reliably identified by traditional culture. Previous work has determined that even in the setting of a CF pulmonary exacerbation, 41% of BALF samples are negative for CF-related bacteria utilizing standard culture [38]. Our findings also confirm the poor sensitivity of traditional culture, especially when there may be a low burden of bacteria present in the airway. Finally, there was significant percent agreement between BALF culture and NP culture, with both standard methods having difficulty detecting the bacteria identified by non-culture based sequencing methods.

What is the clinical significance of microbiota in the CF infant airway? Previous work has demonstrated airway inflammation [39–41], airway infection [41–43], structural lung damage [41,44–49], and decreased lung function [50–53] in infants with CF as young as 4 weeks of age. Remarkably, these studies showed that although inflammation, bronchiectasis and reduced lung function may be present, most infants have no clinically apparent symptoms [39,41,49]. Airway infection is known to play a role in the pathophysiology of CF lung disease. The presence of anaerobic bacteria (*i*.*e*. *Streptococcus*, *Prevotella* and *Veillonella*) may be associated with less inflammation although recent work revealed CF airways infected with anaerobes may contribute to an increased IL-8 through the production of short chain fatty acids [54–56]. The role these bacteria are playing in the development of lung disease remains under investigation. Although our clinical findings were not statistically significant likely secondary to a small number of subjects, the trend we observed of a higher FEV_0.5_ in those CF infants with *Streptococcus* needs to be better defined with further study.

Our work is not without significant limitation. Our cohort of CF infants was small and only three infants received a repeat bronchoscopy at one year of age. However, our descriptive results may be useful to power future studies of microbiota in children. Given the bronchoscopy with lavage was performed in conjunction with the sedation provided for an iPFT, general anesthesia was not used and the bronchoscope was introduced into the infant airway through the nose, increasing the possibility of contamination by the upper airway. We acknowledge that this may not allow us to distinguish between upper respiratory flora contamination and lower airway infection. The presence of *Burkholderia* in our infant BALF samples was initially of significant concern. Although it appears this taxa may be related to background noise, we cannot conclude with certainty that this was the case. Finally, BALF was collected from the middle lobe only, potentially missing bacteria present in additional airways that were not sampled [57,58].

Our study is the first to report on the microbiota of BALF in asymptomatic CF infants. It describes the presence of taxa not detected by traditional culture of either upper or lower airway secretions, highlighting the limitations of current clinical practice. Future study of CF infants is needed to better define the role these taxa, specifically anaerobes, may be playing in the development of CF lung disease.

## Supporting Information

S1 Table Clinical data for each subject at each time point during the study. (DOCX)

## Abbreviations

BALF: Bronchoalveolar lavage fluid
CF: Cystic fibrosis
FVC: Forced vital capacity
FEF: Forced expiratory flow
FEV_0.5_: Forced expiratory volume in 0.5 seconds
iPFT: Infant pulmonary function test
MH: Morisita-Horn
NP: Nasopharyngeal
OTU: Operational taxonomic units
OP: Oropharyngeal
qPCR: Quantitative polymerase chain reaction
RA: Relative abundance
rRNA: Ribosomal RNA
TBL: Total bacterial load
UMN: University of Minnesota
