# Supplementary material for: Airway Microbiota in Bronchoalveolar Lavage Fluid from Clinically Well Infants with Cystic Fibrosis
Source: PLoS One. 2016 Dec 8;11(12):e0167649. doi: 10.1371/journal.pone.0167649 (PMC5145204; doi:10.1371/journal.pone.0167649)
Supplement: S1 Table — (DOCX) [file pone.0167649.s001.docx]

| SUBJECT | GENDER | AGE  (MOS) | GENOTYPE | ANTIBIOTICS | HEIGHT  (CM) | WEIGHT  (KG) | BALF  (%PMNs) | FVC  (%Pred) | FEV_0.5_  (%Pred) |
| --- | --- | --- | --- | --- | --- | --- | --- | --- | --- |
| A | F | 5.7 | F508del/F508del | NA | 65.5 | 8.2 | 20 | 104 | 92 |
| B | F | 5.7 | F508del/F508del | Inhaled Tobramycin (every other month) | 60.3 | 6.8 | 9 | 116 | 94 |
| B | F | 11.9 | F508del/F508del | Inhaled Tobramycin (every other month) | 71.4 | 8.8 |  | 145 | 145 |
| C | F | 6.0 | F508del/F508del | NA | 64.5 | 6.4 | 18 | 88 | 83 |
| C | F | 12.1 | F508del/F508del | Bactrim | 71.5 | 8.9 | 6 | 94 | 97 |
| D | F | 6.1 | F508del/F508del | NA | 66.9 | 7.0 | 4 | 117 | 116 |
| D | F | 13.2 | F508del/F508del | NA | 76 | 8.5 | 7 | 122 | 131 |
| E | F | 6.2 | F508del/F508del | NA | 68 | 8.0 | 16 | 115 | 101 |
| F | M | 6.5 | F508del/F508del | NA | 67 | 8.2 | 15 | 105 | 106 |
| G | F | 6.5 | F508del/F508del | NA | 65.6 | 6.9 | 5 | 133 | 69 |
| H | M | 11.8 | F508del/ G551D | NA | 75 | 9.9 | NA | 96 | 94 |
| H | M | 6.3 | F508del/ G551D | NA | 67.2 | 8 | 1 | 119 | 125 |

**Table S1.** Clinical data for each subject at each time point during the study
